# Hyperexcitability: From Normal Fear to Pathological Anxiety and Trauma

**DOI:** 10.3389/fnsys.2022.727054

**Published:** 2022-08-04

**Authors:** Jeffrey B. Rosen, Jay Schulkin

**Affiliations:** ^1^Department of Psychological and Brain Sciences, University of Delaware, Newark, DE, United States; ^2^School of Medicine, University of Washington, Seattle, WA, United States

**Keywords:** hyperexcitability, neural circuitry, CRF, fear, threat, anxiety, PTSD, somatostatin

## Abstract

Hyperexcitability in fear circuits is suggested to be important for development of pathological anxiety and trauma from adaptive mechanisms of fear. Hyperexcitability is proposed to be due to acquired sensitization in fear circuits that progressively becomes more severe over time causing changing symptoms in early and late pathology. We use the metaphor and mechanisms of kindling to examine gains and losses in function of one excitatory and one inhibitory neuropeptide, corticotrophin releasing factor and somatostatin, respectively, to explore this sensitization hypothesis. We suggest amygdala kindling induced hyperexcitability, hyper-inhibition and loss of inhibition provide clues to mechanisms for hyperexcitability and progressive changes in function initiated by stress and trauma.

## Introduction

In this paper we explore and update the idea that pathological anxiety develops from hyperexcitability of neural circuits for adaptive fear. More than 20 years ago we presented a view that pathological anxiety developed through a process of neural sensitization or kindling-like processes that initiate changes in the brain’s adaptive fear circuits leading to enhanced perception and response to subsequent threat and danger (Rosen and Schulkin, 1998). This not only includes fear-related autonomic and behavioral responses activated during pathological anxiety, but the perceptual fear response of greater vigilance. This hypervigilance is super-responsiveness to events which may be threatening (Frijda, 1986). Unraveling the mechanisms of the perceptual fear response may lead to a greater understanding of pathological anxiety because dysfunction or overactivation of the perception of fear leads to anxious thought and maladaptive behavior.

We use the metaphor and mechanisms of kindling induced-seizure development as a basis for a progression of hyperexcitability in excitatory fear circuits to move from adaptive fear to pathological anxiety and trauma disorders. We extend this idea further to include kindling and sensitization induced hyper-inhibitory and disinhibitory processes interacting with excitatory neurons which produce their own effects in fear circuits and symptoms associated with anxiety and trauma, such as numbness, aloofness, and lack of detail and loss of context in the memories of traumatic and anxious incidents.

Hyperexcitability and hyper-inhibition in neural circuits responsible for normal adaptive fear states and behaviors may lead to exaggeration or persistence of these states and behaviors that behaviorally is seen as maladaptive fear. At the level of neural circuits, the amygdala and its connections are central for both normal fear and pathological anxiety. We suggest that while hyperexcitability in the amygdala is responsible for hypervigilance and exaggerated responses to threat in pathological anxiety, hyper-inhibition, and progressive loss of inhibitory processes in the hippocampus and prefrontal cortex also contribute to the spectrum of symptoms. Kindling-like processes might produce both hyperexcitable and hyper-inhibitory states as adaptation to excessive activation of neural circuits.

## Kindling: A Metaphor for Hyperexcitability

Humans inherently use metaphors as a reasoning tool to make sense of our ourselves and the physical world (Johnson, 1983). The concept of “kindling” began as a metaphor from lighting small twigs and branches of wood to ignite a full-blown fire in larger logs to describe how repetitive low level electrical stimulation in the amygdala or hippocampus develops into full-blown seizures over time (Goddard, 1967; Goddard et al., 1969; Abel and McCandless, 1992). The term kindling was extended to describe the development of chemically induced seizures with repetitive sub-convulsive dosing of drugs like lidocaine, cocaine (Post et al., 1975; Post and Kopanda, 1976), and others (Abel and McCandless, 1992). Nevertheless, we will focus on electrical kindling in this paper.

Electrical kindling can be induced in many areas of the brain, but in most research the amygdala or hippocampus of rodents is stimulated because these regions are typically the loci for temporal lobe epilepsy in humans (Gorter et al., 2016). Kindling starts as a short electroencephalographic afterdischarge and a mild behavioral response (arrest or freezing) to electrical stimulation (typically 1 s in duration). With repeated stimulation for about 2 weeks, a full-blown seizure develops through a very predictable behavioral sequence (McIntyre et al., 2002; Racine et al., 2002; Löscher, 2017). The stimulation parameters do not change, but brain’s response to the stimulation changes. The brain becomes more sensitive to each new stimulation, with a lowering of threshold and a slow development in the severity of neural and behavioral seizure activity. If kindling stimulation is stopped after partial localized seizure activity is induced and then restarted again after a pause of weeks to months, the seizure intensity will begin at the same level as before restimulation and then continue to intensify with more stimulation (Goddard et al., 1969; Pinel and Rovner, 1978; Kalynchuk, 2000). Because of the methodological and sensitizing similarities to long-term potentiation, kindling is suggested to be a model for learning and memory (Goddard and Douglas, 1975; McEachern and Shaw, 1999; Albensi et al., 2007; Meador, 2007).

Although kindling is a model of seizure development, it has fallen out of favor as an epilepsy model in recent years because it does not readily lead to spontaneous seizures that are part of the clinical syndrome of the epilepsies (Gorter et al., 2016; Löscher, 2017; Sutula and Kotloski, 2017). Nevertheless, with more than a hundred daily stimulations spontaneous seizures can develop (Pinel and Rovner, 1978; Milgram et al., 1995; Michael et al., 1998; Sayin et al., 2003; Brandt et al., 2004; Liu et al., 2021). Whether other epilepsy models better capture human epilepsies is disputable (Morimoto et al., 2004; Sloviter, 2005). Still, kindling is most useful as a model of focal non-motor partial seizures (McIntyre, 2006) and to study the progression of seizures and their behavioral consequences, particularly changes in emotionality (Kalynchuk, 2000).

The kindling metaphor is a useful way of thinking about how anxiety, depression and bipolar disorders may develop with repeated exposure to psychosocial stressors (Rosen and Schulkin, 1998; Post, 2002). Robert Post and colleagues proposed a kindling heuristic for anxiety and mood disorders, where with repeated trauma, the individual becomes more sensitive to aversive stimuli and events, and seems primed to develop more and more severe affective psychosis (Post and Kopanda, 1976; Post, 1992, 2002, 2007; Post and Weiss, 1998; Weiss and Post, 1998). Clinically, a kindling-like sequence has been described for depression where depressive episodes beget more depressive episodes. It is estimated that having a single depressive episode leads to a 50% likelihood of having a second episode, after a second episode there is a 70% likelihood of having a third, and then after three episodes it is a 90% likelihood the person will have more episodes (American Psychiatric Association, 2000). Furthermore, once depressive episodes become recurring, less intense external events might trigger new episodes (Association, 2013). Individuals become more sensitive to perturbations in their environment, have excessive intrusive thoughts and musings which may trigger a depressive episode. We originally proposed that anxiety and trauma disorders can be thought of increased hyperexcitability in the amygdala and its circuits (Rosen and Schulkin, 1998) and many studies support this idea (Morgan et al., 1995; Lähdepuro et al., 2019; Gould et al., 2021).

Kindling as metaphor acts as a rubric for many types of experimental paradigms modeling post traumatic stress disorder (PTSD), anxiety disorders and pathological fear. Acute or chronic stress through restraint/immobilization, chronic unpredictable stress, repeated shocks, exposure to predators and predator odors, isolation, single prolonged stress, social defeat, and early maternal care stress are all paradigms that “kindle” fear circuits to become hyperexcitable and more sensitive to the perception of threatening events leading to exaggerated behavioral and psychological responses [for review see (Campos et al., 2013; Miles and Maren, 2019)]. For example, like amygdala kindling, in stress-enhanced fear learning (SEFL), a rodent model of PTSD, a series of repeated foot-shocks enhances subsequent fear responses to threat in contexts unrelated to the foot-shock experience suggesting a mechanism of stress-induced sensitization (Rau and Fanselow, 2009; Perusini et al., 2015; Poulos et al., 2016).

**FIGURE 1:**
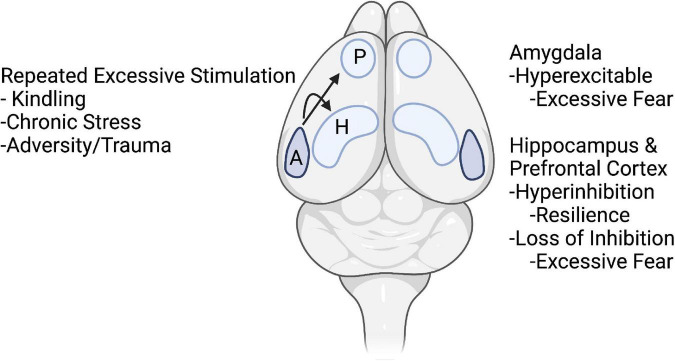
Schematic drawing of topics of the article. It is hypothesized that repeated excessive stimulation of kindling, chronic stress, and trauma initiate changes in nodes of fear circuits. In amygdala, these events lead to hyperexcitability and excessive fear. In the hippocampus and prefrontal cortex, early stages of kindling and acute stress may lead to compensatory hyper-inhibition and resilience, whereas repeated kindling, stress, and trauma lead to loss of inhibition and excessive fear. Arrows from the amygdala (A) indicate excessive activity in the amygdala during kindling, stress, and trauma not only produces hyperexcitability in the amygdala but also causes changes in inhibitory and excitatory function in the hippocampus (H) and medial prefrontal cortex (P). Created with BioRender.com.

In the rest of the paper, we will highlight how information gleaned from kindling might lead to clues about neural mechanisms of hyperexcitability and compensatory changes that may regulate hyperexcitability and hyper-inhibition altering adaptive fear leading toward pathological anxiety. This will be highlighted in the amygdala, hippocampus, and medial prefrontal cortex (Figure 1).

## Kindling of Emotional Hyperexcitability in Fear Circuits

Kindling produces enhanced fear and emotional reactivity in rodents, cats and monkeys during interictal phases, [i.e., between seizures (Adamec, 1990, 2001; Rosen et al., 1996; Kalynchuk, 2000; Lissek and Meurs, 2015)], similar to humans with temporal lobe focal non-motor seizures (Gloor, 1997; Gunn and Baram, 2017). In general, both partial and full kindling produce animals that display more interictal defensive behavior, avoidance, fear and anxiety-like behavior to a number of threatening challenges [reviewed in Kalynchuk (2000), Adamec (2001)]. Thus, it is not the seizure *per se* that is of interest here, but the increased responsiveness to threat and augmented neuronal excitability in the amygdala that the kindling produces (Rosen et al., 1996; Adamec and Young, 2000). Kindling in either the amygdala or hippocampus can induce hyperexcitability with increases in defensive behavior (Kalynchuk, 2000). Amygdala and hippocampal circuitries are more prone to induction of fear hyperexcitability, as caudate nucleus kindling does not facilitate defensive responding to threats (Kalynchuk, 2000). Partial amygdala kindling (without display of overt seizures) also is electrophysiologically confined to circuits related to fear (Rosen et al., 1996; Adamec, 2001). However, once motor seizures begin to develop broader motor circuits connected to the claustrum and/or area tempestas in the pyriform cortex are recruited leading to generalized seizures (O’Shea et al., 2000; Mohapel et al., 2001; Vismer et al., 2015).

## Anxiety and Trauma Disorders Are Not Necessarily a Maladaptation of Fear Conditioning, but Exaggerated Anticipation of Threat

We want to emphasis here that we think trauma and anxiety disorders are not a maladaptation of fear conditioning. The processes and mechanisms of fear conditioning are intact in these disorders. What is changed is a lowering of the threshold for activation of the amygdala and fear circuits in these disorders which can facilitate expression of previously learned fear (Rosen et al., 1996) or enhance anxiety-like and defensive behavior in fear-inducing contexts, like an open field, elevated-plus maze, and a resident-intruder paradigm (Kalynchuk et al., 1999). Animal models of PTSD, like the SEFL and early stress exposure models, also induce sensitization that enhances subsequent fear conditioning and generalization of fear (Rau et al., 2005; Perusini et al., 2015). A kindled individual with an anxiety or trauma disorder should be sensitized and have reduced threshold to respond to and anticipate threats more readily than non-anxiety disorder individuals but have intact fear conditioning abilities. A recent study in anxiety disorder patients demonstrated a bias for anticipating threat, but normal fear conditioning (Abend et al., 2020). Anxiety disorder patients learned a fear conditioning and extinction task comparably to normal subjects as measured by skin conductance. However, the anxiety patients had higher levels of skin conductance at each phase of the experiment, that is, before conditioning, during the fear conditioned test and during extinction. The results suggest that fear conditioning responses are intact, but anxiety patients are in a heightened state of anticipation of threat (Abend et al., 2022).

## Hyperexcitability in the Amygdala Is a Core Feature of Anxiety and Trauma Disorders

From the hypothesis that disorders of anxiety and trauma biologically stem from hyperexcitability of fear circuits, it is no surprise that the amygdala is hyperactivated in these disorders. Neuroimaging studies have quite consistently found exaggerated amygdala activity compared to non-anxiety normal subjects with fMRI, PET and other imaging techniques (Protopopescu et al., 2005; Etkin and Wager, 2007; Nitschke et al., 2009; Shin and Liberzon, 2010; Brühl et al., 2011; Kim et al., 2011; Wingen et al., 2011; Ipser et al., 2013; Mochcovitch et al., 2014; Bas-Hoogendam et al., 2016). This runs across social anxiety, PTSD, and specific phobias, panic disorder, generalized anxiety and obsessive-compulsive disorders, indicating maladaptiveness of hyperexcitability in the neurocircuitry of the amygdala.

Kindling predicts that sensitization and hyperexcitability in anxiety and trauma disorders is maintained for a long time or even permanently. When kindling stimulation is stopped for an extended period and then begins again, the seizure severity remains at the level with little or no regression (Goddard et al., 1969; Pinel and Rovner, 1978; Kalynchuk, 2000). In humans, threat-related amygdala activity appears to be a biomarker to predict future vulnerability to life stressors 1–4 years later (Swartz et al., 2015). Increased volume and activation of amygdala, and reduced volume, but increased activation, of the hippocampus in children exposed to violence might also be a risk factor for later development of PTSD (Rooij et al., 2020). Looking for mechanisms maintaining kindling-induced hyperreactivity might be useful for pointing to mechanisms maintaining hyperexcitability to threats in anxious and trauma disorders.

## Cortical Integration With Amygdala in Fear, Trauma and Anxiety Disorders

Interaction of the prefrontal and cingulate cortices with the amygdala in anxiety and trauma disorders is important because of their role in inhibition and regulation of fear responses. The ventral prefrontal and anterior cingulate cortices are involved in extinction, inhibition and regulation of fear, but also retrieval of fear memories (Etkin et al., 2011; Milad and Quirk, 2012; Do-Monte et al., 2015a,b; Gross, 2015; Kitamura et al., 2017; Quiñones-Laracuente et al., 2021). Meta-analyses generally find that the prefrontal cortex and anterior cingulate cortex are less active in PTSD and generalized anxiety disorder patients than normal subjects (Etkin and Wager, 2007; Wood et al., 2011; Simmons and Matthews, 2012; Mochcovitch et al., 2014). Habituation in the anterior cingulate cortex to fear stimuli shortly after an acute traumatic experience also predicts later PTSD symptoms (Stevens et al., 2016). These regions play a role in regulating expression and long-term memory of fear (Corcoran and Quirk, 2007; Quirk and Mueller, 2008; Etkin et al., 2011; Milad and Quirk, 2012; Kitamura et al., 2017) and are also involved in threat appraisal and evaluation (Etkin et al., 2011). The anterior cingulate and insula cortices are particularly engaged in interoceptive-emotional integration of bodily responses, such as heart-evoked potentials, during a facial emotional recognition task (Salamone et al., 2021).

Other cortical regions, like the insula cortex, which receives interoceptive information from the viscera and body, are also important (Fitzgerald et al., 2018) and have reciprocal connections with the amygdala. The insula cortex is hyperactive in anxiety and trauma disorders to provocation (Fitzgerald et al., 2018). Evidence from kindling studies indicates that development of kindled seizures is first propagated from amygdala to the insula cortex during the early stages of kindling when only arrest/immobile behavior is generated (O’Shea et al., 2000; Foster et al., 2004), suggesting amygdala hyperexcitability readily recruits the insula cortex, which then also becomes hyperresponsive to threatening stimuli.

## Heterogeneity of Hyperexcitability in Anxiety and Trauma Patients

Not all anxiety and trauma patients display a pattern of hyperactive amygdala and hypoactive prefrontal/cingulate cortex. And not all patients are hyper-responsive to perceived threat. About 30% of PTSD patients have the opposite brain activity to threat – hypoactive amygdala and hyperactive prefrontal/cingulate cortex (Lanius et al., 2006). These patients are also different behaviorally, where they report feelings and symptoms of chronic dissociation (aloofness, detachment, depersonalization, de-realization, and subjective distance from emotional experience). This aloofness and detachment are not only found in PTSD patients, but also in social anxiety patients, particularly as they age to become detached and emotionally distant (Schmidt and Poole, 2019).

These anxiety and trauma patients have an over modulation of affect, where the prefrontal/cingulate cortex dampens the activity of the amygdala and insula cortex in response to emotional stimuli (Lanius et al., 2010a,b). Interestingly, in addition to reduced responses to threat, these patients also have dampened responses to happy stimuli (e.g., happy faces). Dissociative PTSD patients typically have a history of early trauma or repeated trauma, whereas non-dissociative, hyper-responsive PTSD patients usually have an acute or single traumatic experience (Lanius et al., 2010a). Early trauma, particularly prepubertal, is associated with less amygdala reactivity to negative content pictures (Zhu et al., 2019; Sicorello et al., 2021). Finally, dissociative symptoms and hyperexcitability/hypervigilance to threat are part of the diagnostic criteria for PTSD and anxiety disorders, and individuals experience both of these states as they attempt to regulate their emotions, where detachment and hypervigilance cycle as various circumstances change throughout the day (Yehuda et al., 2015; Lanius et al., 2017).

Kindling models may be helpful in explaining some of the dampened affect. Kindling initiates neuronal plasticity and hyperexcitability through NMDA and AMPA glutamatergic receptors and calcium channels similar to long-term potentiation (Goddard and Douglas, 1975; Meador, 2007). Hyperexcitability may occur in both excitatory pyramidal neurons and inhibitory interneurons. Hyperexcitability in inhibitory interneurons may dampen excitation of excitatory principal neurons diminishing function in affected circuits, but also may cause disinhibition through increase in inhibition of inhibitory interneurons causing increased excitatory circuit activity. With continued kindling, excessive glutamate release may produce excitotoxic neural degeneration and neuronal loss, and even disuse/disconnection degeneration (McEachern and Shaw, 1999). This may occur in both excitatory and inhibitory neurons (Cavazos and Sutula, 1990; Cavazos et al., 1991; Sayin et al., 2003; Sutula and Dudek, 2007). Kindling- and trauma-induced sensitization and neuronal perturbations are also affected by previous trauma and age, where trauma and aging may compromise maintenance of energy states and membrane potentials allowing for more excitotoxic damage during repeated insults (McEachern and Shaw, 1999), changing the emotional dimensions of anxiety disorders over time.

## Anxiety and PTSD Patients Have Memory Deficits, but Reminisce About Their Anxiety and Trauma: Role of Hippocampus

In addition to being hyperexcitable to threatening and dangerous stimuli, anxiety and PTSD patients have deficits in several memory processes including sematic memory, detail for autobiographical episodic memory, and contextualization of memories [reviewed in (Joshi et al., 2020)]. An underactive hippocampus during memory and extinction tasks may underlie these deficits, and for re-experiencing and reminiscing about prior negative experiences and trauma (Joshi et al., 2020). Reduced hippocampal connectivity with the posterior cingulate cortex (a hub in the default mode network) may play an important role in autobiographical construction and elaboration of memory (Miller et al., 2017; Lanius et al., 2020). While reduced hippocampal volume, shrunken dendritic branching and impaired neurogenesis with stress may play roles in an underactive hippocampus, also increased inhibitory processing may be important in enhancing long-term depression and impairing LTP (Kim and Diamond, 2002; Leuner and Shors, 2013). One inhibitory molecule which may be important is somatostatin, where somatostatinergic neurons in the dentate gyrus have profound effects on synaptic plasticity (Tallent, 2007).

From the kindling metaphor, it is possible that fear situations which induce hyperexcitability in the amygdala also promote inhibitory mechanisms in the hippocampus. Inhibitory LTP can be produced in somatostatinergic interneurons (Udakis et al., 2020) enhancing suppression of excitability in principal neuron dendrites. Increases in inhibitory LTP may decrease the size of hippocampal fear memory engrams (Stefanelli et al., 2016). Excessive kindling-induced inhibition may shrink engram neuronal ensembles sufficiently to interfere with new learning, leaving anxious or traumatized individuals without the ability to adapt and regulate their fear memories. Increased inhibition of hippocampus would promote hippocampal disconnection from the default mode network (Miller et al., 2017; Viard et al., 2019), disengaging episodic, contextual, and working memory processes during the reminiscing of prior anxious and traumatic experiences.

## Hyperexcitability in Amygdala Neurons in Kindling and Stress: Similar Loss of Intrinsic Regulatory Mechanisms of Hyperpolarization

Using the kindling metaphor, a threatening or traumatic episode would cause a strong fearful response followed by reorganization of neural processes and circuits subserving fear. This may include a reduced threshold for excitation and compensatory inhibitory processes to maintain emotional stability during low threat, but increased perception and responding when threat occurs. As more anxious episodes occur, the brain may go through repeated reorganization to maintain stability. Indeed, reorganization of neural circuits is a defining feature of animal paradigms of stress, anxiety, and trauma (Leuner and Shors, 2013). Chronic stress induces hyperexcitability in neurons of the lateral nucleus of the amygdala with a loss of calcium-activated potassium channel hyperpolarization reducing the threshold for action potentials, allowing for more neuronal firing during threat than normal (Rosenkranz et al., 2010). This stress-induced loss of calcium-activated potassium channel hyperpolarization in the basolateral amygdala is similar to what occurs with amygdala kindling (Rainnie et al., 1992).

## Kindling Mechanisms of Hyperexcitability

Neural mechanisms of kindling-induced long-lasting hyperexcitability involve the efficacy of pre-existing excitatory and inhibitory synapses, and structural rearrangements of neurons (McNamara, 1995; Sutula and Kotloski, 2017). These include changes in intrinsic currents, altered gene expression in molecules of synaptic transmission, neurogenesis, cytoskeletal reorganization and biosynthesis, cation transporter and receptor activity (Corcoran et al., 2011; Gorter et al., 2016). Imbalances in excitation versus inhibition in epilepsy and kindling have been difficult to establish (Staley, 2015), although an interplay between these processes is important for development of kindling and effects of interictal emotional behavior.

In the initial phases of kindling, mechanisms of threshold reduction and hyperexcitability are similar to those of LTP (Schubert et al., 2005), a mechanism for fear learning and memory of fear in the amygdala (Maren and Fanselow, 1996; Blair et al., 2001; Rodrigues et al., 2004; Maren, 2005; Schafe, 2005; Sigurdsson et al., 2007; Sah et al., 2008), but then kindling and LTP diverge (Cain, 1989; Cain et al., 1992; Hughes et al., 1999; McEachern and Shaw, 1999; Heida et al., 2009; Gorter et al., 2016).

NMDA receptors in the amygdala are involved in initiation of kindled seizures, but not for maintaining the prolonged interictal behavioral hyperexcitability (McNamara et al., 1988; Adamec, 1997). Expression of NMDA receptor subunits is not altered in the amygdala 14 days after the last amygdala kindled seizure (Corcoran et al., 2011), but AMPA and metabotropic glutamate receptors appear to mediate kindling-induced hyperexcitability in the basal amygdala nucleus (Rainnie et al., 1992; McNamara, 1995; Löscher, 1998), possibly through changes on presynaptic and postsynaptic regulation of glutamate release and postsynaptic responses (Holmes et al., 1996; Neugebauer et al., 1997). Increases in glutamate release promotes discharge bursting in interneurons which preconditions pyramidal neurons to bursting discharges and enhanced release of glutamate (Wang et al., 2021), likely through kindling-induced loss of calcium-activated potassium channel hyperpolarization in basolateral amygdala neurons (Rainnie et al., 1992). One of the longest lasting changes reported after kindling is of proteins of the SNARE complex showing alterations 12-months after kindling, suggesting a long-lasting, even permanent, change in neurotransmitter release mechanisms (Matveeva et al., 2008). Increased expression of synaptic vesicle protein 2, a protein in secretory vesicles following kindling (Matveeva et al., 2012), is particularly interesting since it is a binding target of the anticonvulsant drug levetiracetam (Ciruelas et al., 2019).

Loss of GABAergic neurons and or changes in subunit composition of GABA receptors in the amygdala and piriform cortex may contribute to kindling-induced hyperexcitability (Petrasek et al., 1992; Lehmann et al., 1998; Pitkänen et al., 1998; Corcoran et al., 2011), although not all studies find significant total reductions in GABA receptors in the amygdala (Tuunanen et al., 1997; Corcoran et al., 2011). There is a loss of GABA-stimulated Cl^–^ influx, possibly reducing GABA-mediated inhibition (Sneddon et al., 1990; Tietz and Chiu, 1991). GABA and benzodiazepine agonist effects are potentiated in regions with reduced GABA-mediated inhibition, possibly suggesting compensatory effects, to maintain neuronal homeostasis (Tietz and Chiu, 1991; Petrasek et al., 1992; Staley, 2015).

Neuropeptides, co-localized in GABAergic interneurons, might also be important for regulating hyperexcitability because they are released when neurons fire at high frequency or in bursts, suggesting they modulate excessive activity and subsequent hyperexcitability induced by kindling and stress (Hökfelt et al., 2018).

Indeed, kindling increases or decreases the expression of many inhibitory neuropeptides like neuropeptide Y, somatostatin, and thyrotropin releasing hormone (Schwarzer et al., 1996; Tuunanen et al., 1997; Vezzani and Hoyer, 1999; Gu et al., 2004; Botterill et al., 2015b,a). Some of these have shown to be quite important for regulating kindling and epileptic seizures in humans. Somatostatinergic interneurons play a particularly important role in kindling and epilepsy (Binaschi et al., 2003). Somatostatinergic interneurons are also important for regulating emotional learning, memory, and behavior (Wolff et al., 2014; Stefanelli et al., 2016; Krabbe et al., 2019; Cummings and Clem, 2020), and are reduced in number in postmortem brains of depressed patients (Tripp et al., 2011; Fee et al., 2017). Kindling also increases the expression of the pro-seizure neuropeptide, corticotrophin releasing factor (CRF; Weiss et al., 1986; Kling et al., 1993; Greenwood et al., 1997; Smith et al., 1997). CRF is well known to play a regulatory role in fear, anxiety, depression and stress-induced neuronal sculpting in amygdala, hippocampus, prefrontal cortex and many other brain regions (Chen et al., 2010, 2012; Maras and Baram, 2012; Henckens et al., 2016; Uribe-Mariño et al., 2016; Birnie et al., 2019). In the remainder of the article, we will focus on somatostatin and CRF as examples of kindling mechanisms in the amygdala, hippocampus and prelimbic cortex that may provide clues to how excitatory and inhibitory neuropeptides may regulate transitions from normal fear to pathological anxiety.

## Somatostatin: An Inhibitory Interneuron Regulating Fear, Kindling and Hyperexcitability

Somatostatin has an important and interesting regulatory role in fear, kindling and hyperexcitability induced by stress. In separate literatures of fear, kindling and chronic stress, somatostatin has received substantial attention in regulating fear-related behavior and hyperexcitability, respectively. We will make a case that kindling-induced changes in somatostatinergic neurons may provide clues to both hyperexcitability in the amygdala, and hyper-inhibition and then collapse of inhibition in the prefrontal cortex and hippocampus associated with anxiety and trauma disorders.

One note is that we will focus on somatostatin in the basolateral amygdala. There is an abundant literature on the role of somatostatin in the central nucleus of the amygdala regulating selection of adaptive fear behavior during different threatening situations (Haubensak et al., 2010; Yu et al., 2016; Fadok et al., 2017, 2018; Sun et al., 2020). We limit our discussion to somatostatin in basolateral amygdala because the scant kindling literature finds most of the somatostatin cell number changes are in the basolateral and not central amygdala (Pitkänen et al., 1998). This may change as more research is done on kindling-induced changes in amygdala somatostatin.

## Somatostatin in Fear

Somatostatin is an abundant inhibitory neuropeptide neurotransmitter co-localized in GABAergic neurons. Somatostatin is expressed about 10–17% GABAergic neurons in numerous nuclei of the rat amygdala, (McDonald and Mascagni, 2002; McDonald, 2020; Vereczki et al., 2021), hippocampus, and dentate gyrus GABAergic neurons (Kosaka et al., 1988; Buckmaster and Jongen-Rêlo, 1999), and 30% of GABAergic neurons in the cortex (Lee et al., 2010). Somatostatinergic interneurons are distinct from parvalbumin-expressing and vasoactive intestinal peptide GABAergic interneurons with little to no co-localization (Freund and Buzsáki, 1996; McDonald, 2020). These three types of inhibitory cells work in concert during fear. In general, vasoactive intestinal peptide interneurons act to inhibit both parvalbumin- and somatostatin-expressing neurons causing disinhibition of excitatory principal neurons (Artinian and Lacaille, 2018; Krabbe et al., 2019; Perumal and Sah, 2021). Parvalbumin-expressing neurons synapse on the soma, proximal dendrites and axons of glutamatergic principal neurons to provide strong inhibition on these excitatory cells (McDonald, 2020). Somatostatinergic interneuron axons target distal parts of principal cell dendrites suggesting selective gating of excitatory inputs onto principal neurons (Yang et al., 2016; Kim et al., 2017; Cummings et al., 2021b). Parvalbumin and somatostatinergic interneurons also have different distributions in various parts of the cortex, where parvalbumin interneurons dominate in sensory-motor regions and somatostatinergic interneurons dominate in medial prefrontal and temporal association cortical regions (Kim et al., 2017), suggesting somatostatinergic interneurons play a pivotal role in cortical plasticity, learning and memory in fear circuits. Somatostatinergic interneurons have similar regulatory control of principal neuron dendritic excitability in the prefrontal cortex, hippocampus, dentate gyrus and basolateral complex of the amygdala for fear learning (Lovett-Barron et al., 2014; Wolff et al., 2014; Stefanelli et al., 2016; Cummings and Clem, 2020). Somatostatinergic neuron knock out mice display more fear and depressive-like behavior in several emotionality tests (Liu et al., 2017) suggesting inhibitory regulation of excitatory principal neurons involved in emotional responsivity.

**FIGURE 2:**
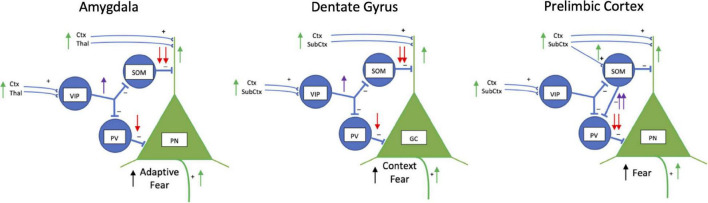
Schematic drawings of inhibitory interneuron regulation of fear in basolateral amygdala, dentate gyrus, and prelimbic cortex. Vasoactive intestinal peptide (VIP), somatostatin (SOM) and parvalbumin-expressing (PV) inhibitory interneurons work together to regulate activity of excitatory pyramidal neurons (PN) in the basolateral amygdala and prelimbic cortex, and granule cells (GC) in the hippocampus. Normally, PNs are strongly inhibited by SOM and PV neurons and fire infrequently (not shown). During fear, in the amygdala and dentate gyrus VIP interneurons (purple arrow) inhibit SOM and PV interneurons (red arrows) which disinhibit PNs (green arrows) activated by conditioned and aversive stimuli signals. In the amygdala and dentate gyrus during fear conditioning, VIP neurons are activated by aversive stimuli (i.e., footshock) through cortical (Ctx), thalamic (Thal) and other subcortical (SubCtx) inputs to strongly inhibit somatostatinergic neurons (SOM) and less strongly inhibit parvalbumin-containing interneurons (PV). Inhibition of SOM interneurons disinhibits dendrites of excitatory PNs and GCs activated during fear. During fear recall, a fear conditioned stimulus alone stimulates VIP interneurons to disinhibit dendrites of PNs and GCs through inhibition of SOM interneurons. In the prelimbic cortex, VIP inhibition appears to play less of a regulatory role. During fear conditioning and recall, SOM interneurons are activated and weakly inhibit PN dendrites, but strongly inhibit PV interneurons (purple arrows) which disinhibit the soma of PNs (red arrows) to facilitate firing of PNs. Disinhibited PNs project to other areas to initiate fear responses. Not shown for simplicity is SOM lateral inhibition of dendrites of nearby PNs. Lateral inhibition limits the number of PNs participating in the fear engram. Also, not shown is reciprocal inhibition between VIP, SOM, and PV interneurons.

Local vasoactive intestinal peptide, parvalbumin and somatostatin GABAergic interneurons mediate a “broad blanket of inhibition” over excitatory principal cells in the cortex, amygdala and hippocampus in non-fear conditioned animals (Karnani et al., 2016). Figure 2 illustrates how a hole in this blanket of local inhibition (Karnani et al., 2016) might “burst” during fear conditioning and recall (Perumal and Sah, 2021). In the basolateral amygdala, excitatory inputs from conditioned (e.g., a tone) and unconditioned (e.g., shock) impinge primarily on the dendrites of principal neurons (McDonald, 2020). They also impinge on inhibitory interneurons. Before fear conditioning, a novel stimulus sends excitatory input from cortex and thalamus to activate principal neuron dendrites which through feedback inhibition to parvalbumin-expressing GABAergic interneurons suppress activity of the principal neuron soma (Unal et al., 2014). At the same time somatostatinergic neurons are also inhibited which disinhibits the principal neuron distal dendrites (Wolff et al., 2014). Input of the unconditioned stimulus synapses on principal neuron dendrites, and synapses on vasoactive intestinal peptide interneurons. Vasoactive intestinal peptide interneurons inhibit both somatostatin and parvalbumin interneurons to disinhibit the principal neuron both at the soma and distal dendrites to enhance associative fear learning (Krabbe et al., 2019). During the expectation of the conditioned stimulus in a recall test, vasoactive intestinal peptide interneurons disinhibit somatostatinergic interneurons to enhance excitation in the principal neuron dendrites promoting neuronal spike generation (Amir et al., 2019; Krabbe et al., 2019). Basolateral amygdala principal cell firing would then activate central amygdala to initiate conditioned fear responses. Although this describes a hierarchical and serial connectivity, it is more complex with reciprocal inhibitory connectivity among vasoactive intestinal peptide, somatostatin and parvalbumin interneurons (Artinian and Lacaille, 2018; Perumal and Sah, 2021).

Similar types of inhibitory-excitatory neuronal interactions occur in the hippocampus and prefrontal cortex (Figure 2). Somatostatinergic interneurons in the hippocampus control the size of the neuronal memory ensembles and affects fear conditioning (Stefanelli et al., 2016). During contextual fear learning a small fraction of excitatory principal neurons in the hippocampus are activated and become part of the memory engram (Ramirez et al., 2013; Tayler et al., 2013). The size of the engram in the dentate gyrus is controlled by lateral inhibition of dendrites of nearby non-active excitatory principal neurons through somatostatinergic inhibitory interneurons (Stefanelli et al., 2016). Inhibiting somatostatinergic interneurons during contextual fear conditioning increases the size of the engram and enhances freezing during recall, whereas activating the somatostatinergic interneurons during learning reduces the size of the engram and impairs recall. Activating or inhibiting parvalbumin-expressing dentate gyrus interneurons has no effect context fear learning (Stefanelli et al., 2016). Thus, similar to gating the strength of auditory fear conditioning in the basolateral amygdala (Wolff et al., 2014), somatostatinergic interneurons in the hippocampus gate spatial learning and contextual fear conditioning.

This schema of gating function by somatostatin in fear learning continues into the dorsomedial (prelimbic) prefrontal cortex (Liu et al., 2017; Cummings and Clem, 2020; Cummings et al., 2021b), however, possibly with different circuitry from that in the amygdala and dentate gyrus (Figure 2). Somatostatinergic interneurons in the prelimbic cortex are activated during fear conditioning and reactivate during memory testing (Cummings and Clem, 2020). Increased activation appears selective to somatostatinergic interneurons, as vasoactive intestinal peptide and parvalbumin interneurons do not display fear conditioning associated Fos activation (Cummings et al., 2021a). Somatostatinergic interneurons activated during fear recall strongly inhibit parvalbumin interneurons synapsing on the soma of principal neurons. This disinhibits the excitatory prelimbic cortical principal neurons to activate their long-range connections to the basolateral amygdala, paraventricular thalamus, ventrolateral periaqueductal gray, lateral habenula and dorsomedial hypothalamus which increases fear responses (Cummings and Clem, 2020).

In addition to classic shock-induced fear conditioning, observational or social fear learning is also regulated by somatostatinergic interneurons in the prelimbic cortex (Liu et al., 2017; Xu et al., 2019). Learning appears to be *via* increasing somatostatinergic activity to inhibit parvalbumin interneurons, disinhibiting excitatory principal neurons (Xu et al., 2019).

## Somatostatin: Kindling-Induced Loss of Somatostatin Inhibition in the Amygdala

A relatively low number of kindling stimulations in the amygdala induces a loss of about 35% of somatostatinergic neurons in the basolateral complex of the amygdala lasting at least 6 months without a significant decrease in total GABA neurons (Tuunanen et al., 1997; Pitkänen et al., 1998). Kindling-induced somatostatinergic cell loss appears to be localized to the basolateral complex as somatostatin-positive neuron number in the central nucleus of the amygdala does not change (Pitkänen et al., 1998). Further, cell loss appears selective for somatostatinergic neurons, since the total number of neurons and overall density of GABAergic neurons were found not to decrease with kindling (Pitkänen et al., 1998). This suggests that selective loss of somatostatinergic inhibition in the basolateral amygdala may increase kindling-induced hyperexcitability (Tuunanen et al., 1997).

**FIGURE 3:**
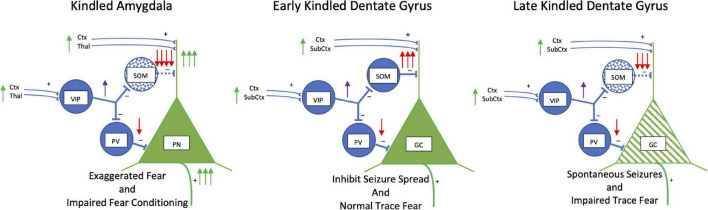
Kindling: Somatostatin. Simplified schematic drawing of the effects of amygdala kindling on somatostatin. In the basolateral amygdala in the left panel, kindling produces a loss of somatostatin interneurons (blue stippling). This releases the glutamatergic principal neurons from strong dendritic inhibition and inhibition at the soma to increase activity of these neurons. This results in exaggerated fear but also an impairment in fear conditioning. In the middle and right panels, the effects of early and late amygdala kindling in the dentate gyrus are shown. During early phases, kindling induces an increase in the number of somatostatin interneurons is induced which strongly inhibits dendritic activity. The strong inhibition reduces the spread of kindled seizures, but trace and context fear conditioning persist. However, during late amygdala-kindling (∼100 stimulations), somatostatin interneurons die, and spontaneous seizures develop. There is a mix of dendritic retraction, loss of spines, and an increase in axonal grow in granule cells (green hatching). Function of the hippocampus is altered leading to impairment of trace and context fear conditioning. Abbreviations are the same as in Figure 2.

The kindling-induced effects suggest a mechanism for exaggerated fear that we propose is core to anxiety and trauma disorders (Figure 3). The loss of somatostatinergic inhibitory interneurons would disinhibit principal neurons increasing their excitability and sensitivity. Mild threats would impinge on the disinhibited basolateral amygdala excitatory principal neurons increasing the probability of activating a cascade of events throughout the connected network to enhance the perception of and responses to fear.

## Hippocampus: Kindling-Induced Gain of Somatostatin Hyper-Inhibition and Then Collapse

Figure 3 also shows the effects of kindling on inhibitory circuitry and somatostatin in the hippocampus. In contrast to the amygdala, somatostatin expression and cell number are increased in the CA1, CA3 of the hippocampus and hilus of the dentate gyrus with amygdala or hippocampal kindling (Shinoda et al., 1989; Schwarzer et al., 1996; Dalby et al., 1998; Vezzani and Hoyer, 1999; Binaschi et al., 2003; Botterill et al., 2017). This is selective for somatostatinergic interneurons as there are small or no changes in the total number of GABAergic or parvalbumin-GABAergic neurons (Botterill et al., 2017). Pharmacologically, somatostatin, somatostatin agonists, and sustained adeno-associated viral mediated somatostatin expression in the dentate gyrus suppress development and expression of kindling and kindling-induced neurogenesis of excitatory granule cells (Vezzani and Hoyer, 1999; Zafar et al., 2012; Natarajan et al., 2017; Leibowitz et al., 2019). Endogenous somatostatin expression increases as kindling proceeds from partial to full kindling (Shinoda et al., 1991) and both basal and stimulated somatostatin release in the dentate gyrus is enhanced following kindling (Marti et al., 2000). This suggests the increase in somatostatin is acting to compensate and control kindling-induced excitability of dentate gyrus glutamatergic neurons driving seizures (Vezzani and Hoyer, 1999; Binaschi et al., 2003). Indeed, somatostatinergic compensation falters when extensive kindling produces spontaneous seizures or in models of status epilepticus (Gorter et al., 2016; Sutula and Kotloski, 2017), a time when the number of somatostatinergic interneurons in the hilus of the dentate gyrus declines (Sayin et al., 2003).

The loss of somatostatinergic interneurons in the dentate gyrus and CA1 is highly selective with 83% of cell loss being somatostatinergic neurons and no loss of VIP-containing interneurons (Buckmaster and Jongen-Rêlo, 1999; Wyeth and Buckmaster, 2021). Surviving somatostatinergic hilar interneurons enlarge, sprout axons and form new synapses on glutamatergic granule cells suggesting somatostatinergic interneurons compensate for the loss of vulnerable interneurons by becoming hyper-inhibitory (Zhang et al., 2009). However, the remaining somatostatinergic interneurons cannot fully compensate for the cell loss (Hofmann et al., 2016), so spontaneous seizures persist. Kindling also induces a mix of apoptosis and neurogenesis in granules cells (Bengzon et al., 1997), with dendritic spine reduction and increases in granule cell body thickness and axon proximal area (Singh et al., 2013). Significant axonal sprouting in the mossy fibers of the granule cells is also a signature of kindling (Sutula and Kotloski, 2017).

Whereas amygdala or hippocampal kindling increases unconditioned and already learned fear, kindling disrupts subsequent fear conditioning (Fournier et al., 2013; Botterill et al., 2014). The severity and chronicity of kindled seizures affect fear learning and memory and neuronal activity in the amygdala and dentate gyrus during trace and delayed fear conditioning differently (Botterill et al., 2014). The amygdala is essential for both delayed and trace fear conditioning, while the hippocampus is important for trace fear learning and memory (Fanselow and Poulos, 2005). Both shorter-term and longer-term amygdala-kindling (30 vs. 99 kindled seizures) disrupts learning and memory of standard delay fear conditioning equally. However, in trace conditioning only longer-term kindling impairs memory retrieval. These behavioral deficits parallel decreases in Fos activity in a region-specific manner. Impaired delayed fear conditioning correlates with decreases in Fos expression in both the hippocampus and amygdala. On the other hand, decreases in trace conditioned fear from longer-term kindling correlates with decreases of Fos in hippocampus, but not amygdala. This study (Botterill et al., 2014) suggests hippocampus function for trace fear conditioning is affected by the severity of kindling, whereas the amygdala is more sensitive to less kindling. Linking this metaphorically to anxiety and trauma, it suggests that the learning and memory functions of the amygdala are readily impaired by less severe and chronic traumatic insults, while the negative effects on the hippocampus occur only with chronic insults.

Whether kindling-induced changes in dentate gyrus somatostatinergic interneurons are involved impairment of trace fear conditioning with long-term kindling is not known yet.

## Somatostatin Interneurons in Acute and Chronic Stress Paradigms

Whereas kindling has not addressed the role of chronicity on somatostatinergic interneuron changes and emotional function, there is a sizable literature on the role of somatostatinergic interneurons in chronic stress paradigms in rodents used to model major affective disorders and PTSD by inducing increases in anxiety-, trauma-, and depressive-like behaviors (Antoniuk et al., 2018; Zhang et al., 2019; Khan et al., 2020). Most research has focused on changes in glutamatergic principal neurons with various repeated and chronic stress paradigms producing neuronal hypertrophy in the amygdala and hypotrophy in the hippocampus and prefrontal cortex (Vyas et al., 2002; McEwen et al., 2012; Moench and Wellman, 2015; Musazzi et al., 2015; Wilson et al., 2015; Cameron and Schoenfeld, 2018; Nikolova et al., 2018; Ortiz and Conrad, 2018; Patel et al., 2019; Woo et al., 2021).

**FIGURE 4:**
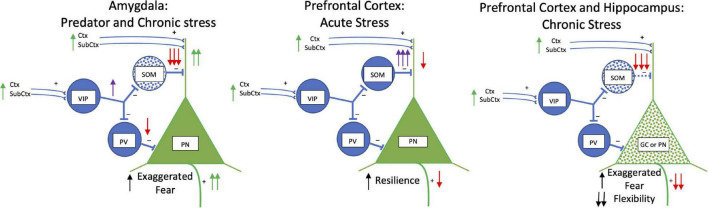
Acute and Chronic Stress: Somatostatin. Simplified schematic drawing of the effects of acute and chronic stress on somatostatin. In the basolateral amygdala in the left panel, predator exposure and chronic stress produce a loss of somatostatin interneurons (blue stippling). This releases the glutamatergic principal neurons from strong dendritic inhibition and inhibition at the soma to increase activity of these neurons. This results in exaggerated fear. In the middle and right panels, the effects of acute and chronic stress in the medial prefrontal cortex and hippocampus are shown. With acute stress there are increases in activity and hyper-inhibition in somatostatin interneurons in the medial prefrontal cortex which strongly inhibits dendritic activity and produces resilience to stress. With chronic stress somatostatin interneurons die (blue stippling) in both medial prefrontal cortex and hippocampus. What results are dendritic retraction and loss of spines in principal and granule cells (green stippling) creating exaggerated fear and loss in cognitive flexibility. Abbreviations are the same as in Figure 2.

Stress studies are beginning to examine GABAergic interneurons, particularly somatostatinergic interneurons (Lin and Sibille, 2015; Fee et al., 2017; Fogaça and Duman, 2019; Figure 4). Various acute and chronic stress paradigms produce different outcomes on principal cells and interneurons likely related to types and duration of stressors (Wilson et al., 2015; Sanacora et al., 2022). Focusing on the amygdala in a single unconditioned predator fear/stress session model of PTSD (Parsons et al., 2018; Albrechet-Souza and Gilpin, 2019), predator odor elicits fear and decreases somatostatinergic interneuron Fos activation in the basolateral and central amygdala (Butler et al., 2011, 2012). More chronic stress (21-day chronic restraint stress) induces dendritic atrophy in GABAergic interneurons (most likely somatostatinergic) in the basolateral amygdala complex (Gilabert-Juan et al., 2011). In another chronic anxiety model, somatostatin receptor 2 gene is reduced (Gaskins et al., 2021). These results parallel the effects of kindling on basolateral amygdala somatostatinergic interneurons producing hyperexcitability (Tuunanen et al., 1997).

Chronic stress generally produces decreases in somatostatinergic cell number and biomarkers in hippocampus and prefrontal cortex (Czéh et al., 2015; Banasr et al., 2017; Girgenti et al., 2019; Newton et al., 2021; Ortiz et al., 2021), but not in all studies (Gilabert-Juan et al., 2013; Fuchs et al., 2016; Gilabert-Juan et al., 2016; Czéh et al., 2018; Moench et al., 2020). This may be related to the chronicity of the stress. For example, chronic stress increases emotionality in a sex selective manner in open field and forced swim tests and impairs cognitive flexibility in an extradimensional shift test (Girgenti et al., 2019; Moench et al., 2020), which is associated with transcriptome differences in somatostatinergic interneurons in the medial prefrontal cortex (Girgenti et al., 2019). Increasing somatostatinergic neuronal activity in the medial prefrontal cortex by deleting γ2 GABA subunits on somatostatinergic interneurons confers resilience to chronic mild stress in male mice (Jefferson et al., 2020). Acute stress produces the opposite effect of chronic stress where prefrontal cortex somatostatinergic interneuron activity is enhanced and becomes hyperexcitable by acute restrain stress through input from the basolateral amygdala (Joffe et al., 2022). Acute stress increases somatostatinergic inhibition of excitatory principal neurons in the medial prefrontal cortex and blocking acute stress-induced somatostatinergic inhibition impairs fear conditioning and recall (Joffe et al., 2022).

Together, results from acute and chronic stress studies parallel the effects of kindling. Both types of stress reduce somatostatinergic interneuron activation in the amygdala, and in the hippocampus and prefrontal cortex somatostatinergic interneuron activity increases or decreases depending on the severity of seizures or chronic stress. This suggests that the reduction of somatostatinergic tone in the amygdala disinhibits principal cell dendrites increasing their excitability to incoming excitatory input. Somatostatinergic enhancement of inhibition on hippocampal and prefrontal cortical principal cell with acute or mild stress would enhance resiliency for fear and anxiety, but with severe chronic stress the loss of somatostatinergic tone would disinhibit principal neurons and induce hyperexcitability.

## CRF: An Enhancer of Kindling and Fear-Induced Hyperexcitability

CRF is involved in many processes in the brain (Schulkin, 2017). It promotes fear, hyperreactivity, defensive aggression and a kindling-like seizure on its own. Single intraventricular injections of CRF produce a slow evolving “kindling-like” sequence of fear and defensive behaviors that ends in a motor seizure after several hours (Ehlers et al., 1983; Baram and Schultz, 1991; Clark et al., 1991). CRF-seizures and amygdala kindling have related mechanisms. Repeated infusion of intraventricular CRF induces the same fear-seizure sequence and facilitates the development of amygdala-kindled seizures (Weiss et al., 1986), indicating CRF-induced seizures initiate sensitization to the electrical kindling stimulation. During early phases of CRF-induced seizures when animals exhibit increases in fear and anxious behavior, the amygdala, insular and pyriform cortices are activated (as measured by c-fos expression), but later, when motor seizures develop the dentate gyrus becomes active (Clark et al., 1991). With amygdala kindling, CRF mRNA expression is increased in GABAergic interneurons of the dentate gyrus (Smith et al., 1997). Whether this *de novo* CRF expression in the dentate gyrus contributes to the development of motor seizures is not known.

**FIGURE 5:**
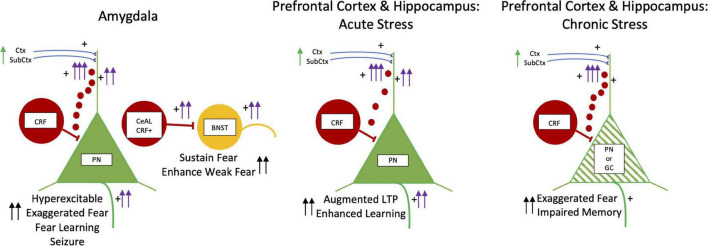
Kindling, Fear, and Acute and Chronic Stress: CRF. CRF neurons are illustrated by red cells and the small red circles depict volume conduction of CRF molecules to dendrites of PN and GC dendrites. BNST is in yellow. PN and GC are in green. The left panel has two schematic drawings of CRF in the basolateral amygdala on the left and the lateral division of central amygdala CRF pathway (CeAL CRF +) projecting to the BNST on the right. CRF is excitatory and infusions induce hyperexcitability in PNs with exaggerated fear, enhanced fear learning, and late onset seizures. In the central amygdala to BNST circuit, CRF is involved in facilitating fear to weak threatening stimuli and sustaining fear long beyond the duration of fear stimuli. In the middle and right panels, the effects CRF in acute and chronic stress in the medial prefrontal cortex and hippocampus are shown. With acute stress CRF enhances learning and augments LTP. With chronic stress, more CRF released in both medial prefrontal cortex and hippocampus. This results in dendritic retraction and loss of spines in principal and granule cells (green hatching) creating exaggerated fear and impaired memory function. Abbreviations are the same as in Figure 2.

Figure 5 summarizes the effects of kindling, and acute and chronic stress of CRF. In the amygdala, CRF is produced primarily in central amygdala GABAergic neurons, but there are also CRF-containing neurons in the basolateral amygdala, which may be glutamatergic (Itoga et al., 2019). CRF1 receptors are highly abundant the basolateral amygdala (Pett et al., 2000; Agoglia and Herman, 2018). It is not clear what the source of CRF is that activates CRF1 receptors in the basolateral amygdala – it can be from volume conduction from the central amygdala or from CRF brainstem projections (Calakos et al., 2016). CRF activates glutamatergic principal neurons and various types of peptide-GABAergic interneurons in the basolateral amygdala through CRF1 receptors which also regulate neuronal responses to chronic unpredictable stress and induce hyperexcitability (Rainnie et al., 2004; Sandi et al., 2008; Ugolini et al., 2008; Rostkowski et al., 2013; Calakos et al., 2016). CRF and CRF1 receptors in the basolateral amygdala facilitate fear, anxiety-like behavior, and fear memory consolidation (Roozendaal et al., 2002; Rainnie et al., 2004; Hubbard et al., 2007).

In the hippocampus CRF-producing cells are co-localized in parvalbumin-containing GABAergic interneurons with their axon terminals surrounding excitatory pyramidal cell bodies, but not somatostatin/calbindin neurons (Chen et al., 2012). CRF through CRF1 receptors augments stress-related learning and memory processes, but also facilitates stress-induced neuronal dysfunction in hippocampus and other brain regions. During short term fear, CRF released by GABAergic/CRF axons at the principal cell soma diffuses to CRF1 receptors on dendrites *via* volume conduction to augment glutamatergic-mediated LTP and enhance memory (Chen et al., 2012). With subacute and chronic stress, prolonged CRF release leads to retraction of dendritic spines on excitatory principal neurons, resulting in loss of LTP and substantial memory impairments (Chen et al., 2010, 2012). In the medial prefrontal cortex, social defeat-induced impairment of working memory is rescued by deletion of CRF1 receptors and mimicked by CRF infusion (Uribe-Mariño et al., 2016). Together stress-induced increase in CRF release produces dendritic damage of principal neurons in both hippocampus and medial prefrontal cortex (Uribe-Mariño et al., 2016). CRF regulated neuronal damage (cortical thinning) of the medial prefrontal and temporal cortices occurs early during stress induced prenatal CRF exposure and has lasting cognitive and emotional deficits seen in early school years (Sandman et al., 2018).

Kindling induces changes in signal transduction of CRF1 receptors and excitability. Kindling generates a switch from CRF being inhibitory to excitatory in the pyriform cortex (Narla et al., 2016). This change is due to a switch from CRF1 receptor signaling through a Ga_q/11_-protein mediated pathway to a Ga_s_-protein pathway. This is through suppression of regulator of G protein signal protein 2 (RGS2), which normally inhibits Ga_s_ mediated signaling through CRF1 receptors. Reducing RGS2 would increase CRF1 receptor linked signal transduction of cAMP, enhancing neuronal hyperexcitability of neurons with CRF1 receptors.

## Corticotrophin-Releasing Factor in the Extended Amygdala: Hyperexcitability, Kindling and Fear

An important CRF pathway for fear is from the lateral division of the central nucleus of the amygdala (CeAL) to the bed nucleus of the stria terminalis (BNST, Figure 5). While this CRF pathway has not been selectively investigated in kindling hyperexcitability, early studies of lesions of the stria terminal pathway facilitate amygdala kindling (Engel and Katzman, 1977; Racine et al., 1988), and amygdala lesions retard kindling from the BNST (Salle, 1979). This suggests that the BNST has an inhibitory influence on amygdala kindling and the amygdala modulates kindling in the BNST. While this earlier kindling work on the amygdala and BNST does not say much about the amygdala-BNST CRF pathway on kindling hyperexcitability, the two regions are linked bidirectionally by GABAergic inhibitory neurons co-localized with numerous peptides (Cassell et al., 1999).

The BNST plays a central role in fear. Early ideas suggested that the central amygdala mediated phasic, short lasting fear responses, while the BNST mediated sustained fear behavior, similar to some views on the distinction between fear and anxiety (Walker and Davis, 2000, 2008; Walker et al., 2009; Davis et al., 2010). More recent experiments and theorizing suggest that the BNST is particularly involved in regulating fear during times of unpredictable threat (Goode and Maren, 2017; Goode et al., 2019, 2020), and there is little distinction between the activity of the amygdala and BNST in fear and anxiety (Gungor and Paré, 2016; Shackman and Fox, 2016; Fox and Shackman, 2019).

CRF containing neurons in the lateral division of the central nucleus of the amygdala which project to the BNST appear to be important for sensitizing responses to threatening situations and stimuli (Walker et al., 2009; Sanford et al., 2016). Similar to hippocampus and medial prefrontal cortex, CRF extended amygdala neurons are co-localized in GABAergic inhibitory neurons, which differ from hypothalamic CRF neurons co-localized with the excitatory neurotransmitter glutamine (Dabrowska et al., 2013; Partridge et al., 2016). Deletion of the GABAα1 subunit in the CRF amygdala-BNST neurons disinhibits these CRF neurons and increases anxious behavior and a persistent deficit in fear extinction (Gafford et al., 2012).

CRF in the amygdala-BNST pathway also facilitates fear learning to weak aversive stimulation (Sanford et al., 2016). Chemogenetic inhibition of CRF in the central nucleus of mice reduces fear learning to a tone paired with weak aversive shock. If the shock is increased to a moderate level, inhibiting these CRF neurons has no effect. These results and results from another study which continuously enhanced CRF (Sink et al., 2013) suggest that these CRF neurons in the amygdala amplify the ability to acquire fear. These CRF neurons act as a hyperexcitability gating mechanism between the lateral nucleus neurons which receive sensory information to the output neurons of the medial division of the central amygdala neurons producing enhanced fear and anxiety (Sanford et al., 2016).

Pharmacological studies antagonizing CRF receptors in the bed nucleus of the stria terminalis and inhibition of CRF production in the lateral division of the central nucleus of the amygdala block the retention of contextual fear memory (Pitts et al., 2009; Davis et al., 2010; Pitts and Takahashi, 2011; Asok et al., 2016; Partridge et al., 2016). Optogenetic silencing of CRF cell bodies in the lateral division of the central nucleus of the amygdala or the CRF axon terminalis of these neurons in the BNST during the learning of contextual or tone fear conditioning reduces the sustained fear behavior in a memory test one day after learning (Asok et al., 2017). Freezing was normal compared to a control group for the first 6 min of the memory test, but then decreased precipitously. Another optogenetic study found that activating these CRF neurons reduced extinction and prolonged freezing (Jo et al., 2020). These studies show how CRF in this extended amygdala-BNST pathway facilitates weak learning, prolongs and sustains fear, and counteracts extinction Schulkin et al., 2005). This CRF pathway may be integrated into an active role for BNST (through connections to the infralimbic cortex) to increase learning and sustain fear when situations are unpredictable and low in threat imminence (Goode et al., 2019, 2020).

## Genetic Influences on Hyperexcitability

Not everyone who experiences severe fear and trauma develops anxiety or trauma disorders. Only about 19% of Vietnam veterans developed PTSD and 11% still have PTSD symptoms 40 years later (Marmar et al., 2015). There is a 50% lifetime prevalence of PTSD in sexually assaulted women (Chivers-Wilson, 2006). This suggests there are biological and gene risk factors for developing of anxiety and trauma disorders.

The analysis of genes and gene mutations associated with anxiety and trauma disorders are still in its infancy, but genes associated with kindling discussed in this paper (CRF and somatostatin) are showing up in these analyses. Genetic polymorphisms associated with anxiety and trauma disorders are found in the glucocorticoid receptor (Rossum et al., 2003; Hauer et al., 2011; Yehuda et al., 2016), genes that regulate the glucocorticoid receptor [e.g., FK506 binding protein 5 (*FKBP5*)] (Binder et al., 2008; Jovanovic and Ressler, 2010; Castro-Vale et al., 2016; Watkins et al., 2016; Banerjee et al., 2017) and genes that regulate the CRF1 receptor (e.g., *SATB1*, a global regulator of gene expression including CRF_1_ receptor (Levey et al., 2020) and pituitary adenylate cyclase peptide and its receptor (*ADCYAP1* and *ADCYAPR1*; Ressler et al., 2011).

GABAergic and somatostatinergic genes also have a genetic association with anxiety and affective disorders. There is an association between glutamic acid decarboxylase (the glutamate to GABA converting enzyme and anxiety and depression (Hettema et al., 2006; Tripp et al., 2012) and an association of GABA activity in prefrontal and cingulate cortices with PTSD (Rosso et al., 2014, 2021). More specifically to somatostatin, there is reduced mRNA expression of somatostatin in lateral and basomedian nuclei of the amygdala, and in the anterior cingulate cortex in postmortem brains of depressive patients (Tripp et al., 2011, 2012; Guilloux et al., 2012). Many of these associated changes GABA, somatostatin and other genes in depression display sex differences (Seney et al., 2021).

Genetic strains of rats that kindle fast or slow have been developed by selective breeding to assess genetic predispositional contributions to acquiring epilepsy and reactivity to uncertainty and threat (McIntyre et al., 2002; McIntyre and Gilby, 2007; Leung et al., 2019). Initial afterdischarge thresholds are not different between fast and slow kindling rats but fast kindling rats develop generalized motor seizures about 4 times as fast as slow kindling rats (Racine et al., 1999). They also have slower and smaller amplitude inhibitory postsynaptic currents in the amygdala than slow kindling rats (McIntyre et al., 1999, 2002). The contrasts may be due to differences in expression of GABA receptor subunits in the lateral and basal amygdala nuclei in slow and fast kindling rats (Poulter et al., 1999). There are also differences in pyramidal cell morphology in the prefrontal cortex between the two strains, but how this relates to seizure susceptibility and behavioral differences in not known (Reinhart et al., 2021). Behaviorally, slow kindling rats display more fearful behavior in an elevated plus maze, open field if previously shocked, and more freezing in inhibitory avoidance, whereas fast kindling rats are hyperactive, impulsive and have deficits on several cognitive tests (Mohapel and McIntyre, 1998; Gilby and O’Brien, 2013). The data suggest slow kindling rats have a genetic predisposition to be more fearful but are more resistant to developing generalized motor seizures. Whether amygdala and/or prefrontal cortex somatostatinergic interneurons play a role in innate behavior differences between these strains has not yet been explored.

One study examined the release of CRF to stressors in fast and slow kindling rats (Merali et al., 2001). Predator (ferret) odor exposure and immobilization stress induces greater CRF in the hypothalamus in slow kindling rats. CRF is differentially regulated in the central amygdala nucleus. Predator odor increases more CRF in slow kindling rats, but immobilization stress elicits more in fast kindling rats. How CRF differentially affects behavior is not known.

## Conclusion

We have explored kindling-induced hyperexcitability and hyper-inhibition as a metaphor for development of pathological anxiety and trauma disorders from adaptive neurocircuits of fear. Kindling induces hyperexcitability in the amygdala which affects many cortical and subcortical regions of the temporal lobe (O’Shea et al., 2000). This is partially by a loss of somatostatin neurons and a compensatory hyper-inhibition of somatostatin neurons in the dentate gyrus that constrains the development of generalized motor seizures. Kindling as a metaphor for development of anxiety disorders suggests somatostatin plays important regulatory roles in anxiety disorder-related phenomena of hyperexcitability in the amygdala to threat and a dampening of hippocampal and medial prefrontal cortex function. We suggest these alterations in somatostatinergic neurons in the amygdala, hippocampus, and prefrontal cortex might regulate fear-related functions in human anxiety and trauma disorders. Gleaning information from kindling-induced changes in somatostatin may point to new directions to investigate somatostatin in animal models of these disorders.

One is that through kindling-like or fear induced non-associative sensitization processes, and not fear conditioning *per se*, maladaptive anxiety becomes triggered by lowering the threshold for threatening stimuli to activate amygdala fear circuitry. Loss of inhibitory somatostatin interneurons in basolateral amygdala may play a large role in this. Increases in CRF excitation, both in the basolateral and central amygdala and BNST further facilitates sensitization. Other inhibitory and excitatory neuropeptide systems also likely contribute.

These kindling-induced changes over time may be similar to what trauma and stress produce. This is reminiscent of the effects of few versus many traumatic episodes in PTSD patients where hypervigilance may change to aloofness and detachment with more trauma episodes (Lanius et al., 2010a). While kindling produces seizures, but fear and trauma do not, we have emphasized mechanisms of fear hyperexcitability which may be similar and what is learned from kindling. This may help guide research into processes that contribute to turning normal adaptive fear into pathological fear and anxiety.

## Editor’s Note

Arun Asok edited the article in collaboration with Eric R. Kandel, Columbia University, United States.

## Author Contributions

JR and JS contributed equally to the conception of the manuscript. JR wrote the first draft. Both authors contributed to the manuscript revision, read, and approved the submitted version.

## Conflict of Interest

The authors declare that the research was conducted in the absence of any commercial or financial relationships that could be construed as a potential conflict of interest.

## Publisher’s Note

All claims expressed in this article are solely those of the authors and do not necessarily represent those of their affiliated organizations, or those of the publisher, the editors and the reviewers. Any product that may be evaluated in this article, or claim that may be made by its manufacturer, is not guaranteed or endorsed by the publisher.
